# Moyamoya-like vasculopathy associated to MYH9-related thrombocytopenia manifested by multiple cerebral ischemic lesions: a case report

**DOI:** 10.1186/s12883-020-01927-6

**Published:** 2020-09-19

**Authors:** Athena Cristina Ribigan, Raluca Stefania Badea, Alida Ciocan, Dana Stefan, Bogdan Casaru, Patricia Ioan, Florina Antochi, Ovidiu Băjenaru

**Affiliations:** 1 Neurology Department, University Emergency Hospital Bucharest, Splaiul Independentei, number 169, district 5, 050098 Bucharest, Romania; 2 Department of Clinical Neurosciences, University of Medicine and Pharmacy Carol Davila Bucharest, Dionisie Lupu street, number 37, district 1, 020021 Bucharest, Romania; 3 Transcranial Doppler Monitoring and Neurosonology Laboratory, Romanian Academy, Bucharest, Splaiul Independentei, number 169, district 5, 050098 Bucharest, Romania

**Keywords:** Moyamoya, Vasculopathy, MYH9, Thrombocytopenia, Ischemic lesions

## Abstract

**Background:**

Moyamoya-like vasculopathy (MMV) and myosin heavy chain 9-related platelet disorders (MYH9-RPDs) or macrothrombocitopenias are rare syndromes. Their association is even more infrequent.

**Case presentation:**

A 29-year-old female with history of MYH9-RPD, presented to our department for episodes suggesting transient ischemic attacks. Based on the imaging studies that revealed multiple ischemic lesions and stenoses of both distal internal carotid arteries and the arteries of the circle of Willis, the diagnosis of MMV was established. The treatment with Verapamil was initiated, leading to symptom remission. Two months later, the patient presented one episode of dysarthria, followed by involuntary movements of the right upper limb, few days later. Long-term electroencephalogram monitoring depicted epileptiform abnormalities. Resolution of symptoms was obtained after increasing the dose of Verapamil, and initiating Levetiracetam.

**Conclusions:**

This is an interesting case of a patient with two rare pathologies, who presented with cerebral ischemic strokes. To our knowledge there are few cases described in the literature presenting with cerebral hemorrhagic events but none of them with multiple cerebral ischemic lesions. As these cases are very rare, it is important to gather evidence regarding the best approach and treatment strategy.

## Background

Moyamoya disease (MMD) is an idiopathic disorder defined by progressive occlusion or stenoses of the intracranial distal segments of the internal carotid arteries (ICAs) and the arteries of circle of Willis associated with the development of numerous collaterals suggesting an aspect of “a hazy cloud like a puff of cigarette smoke” [1]. When the aforementioned abnormalities are present in patients with other concomitant diseases, the condition is called Moyamoya syndrome (MMS) or MMV due to the fact that the underlying disease may participate to the pathogenesis of the vasculopathy. Some of the conditions associated with MMS include hematologic disorders [2].

MYH9-RPD represent a group of hereditary macrothrombocytopenias characterized by thrombocytopenia, giant platelets, and a combination of leukocyte inclusion bodies, hearing loss, nephritis or cataract [3].

The aim of this paper is to present an uncommon case of a young patient with medical history of a rare hematologic disorder namely MYH9-related macrothrombocytopenia, who was diagnosed with MMV. To establish the etiology of the patient’s symptoms extensive laboratory tests were performed. In conclusion, it was certified that the MMS presented with multiple cerebral ischemic lesions. This triad of MYH9-related macrothrombocytopenia – MMV – multiple cerebral ischemic lesions was not previously described.

## Case presentation

A 29-year-old female presented to our Neurology Department for episodes of paresthesias, motor weakness and dysarthria lasting for an hour to few hours. Symptoms onset was 1 month and a half prior to admittance. The episodes affected either the left or the right side of the body and face. The frequency of the episodes varied from one in a few days to three episodes daily. First event appeared during an episode of severe diarrhea with secondary dehydration.

The patient was a smoker, with a family history of MYH9-related thrombocytopenia (mother and eight maternal relatives), thrombophilia and other autoimmune disorders. She had a medical history of thrombocytopenia and hearing impairment (similar with her mother). She was diagnosed with MYH9-RPD 3 years before the admission to our clinic. The general and neurologic exams were normal except for the hearing loss and numerous cutaneous hematomas.

Laboratory findings including tests for autoimmune disorders, vasculitis and thrombophilia were normal with the exception of thrombocytopenia (9000–37,000/μl) and increased titer of anti-thyroid peroxidase antibodies (with normal thyroid function tests). Peripheral blood smear did not reveal any evidence of sickle cell disease or other hematologic disorders, and the patient did not present symptoms suggestive for such a disease. Lumbar puncture was not performed due to low platelet count.

**Fig. 1:**
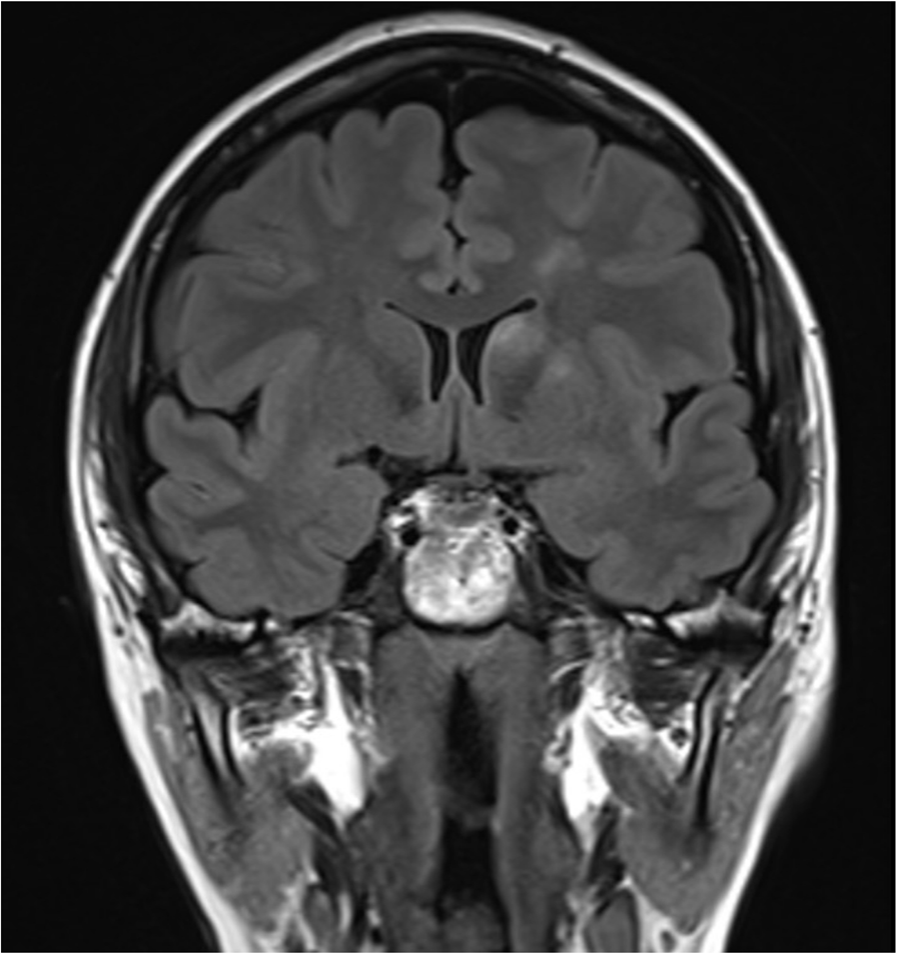
3 Tesla Cerebral MRI coronal FLAIR sequence showing the largest ischemic lesions located in the left cerebral hemisphere

Cerebral magnetic resonance imaging (MRI) depicted the presence of multiple oval or triangular hyperintensities in T2 and FLAIR sequences located in both hemispheres, the cortical-subcortical ones with diffusion restriction in DWI sequences (Fig. 1). Most of the lesions were gadolinium-enhancing. One of the lesions, located in the left head of caudate nucleus showed signs of petechial haemorrhagic transformation in susceptibility weighted imaging.

**Fig. 2:**
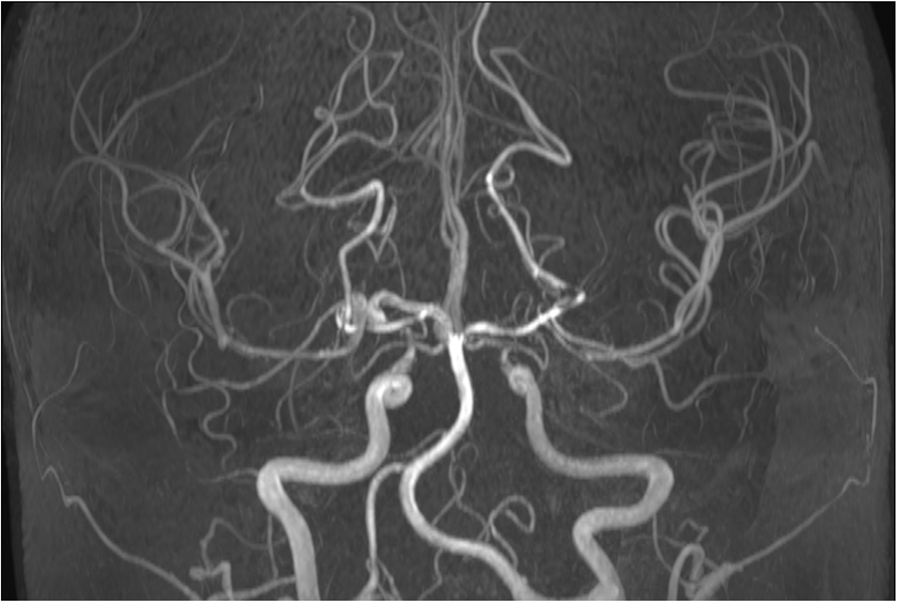
MRA coronal sequence revealing stenoses of both intracranial ICA and also of ACA, MCA and PCA

**Fig. 3:**
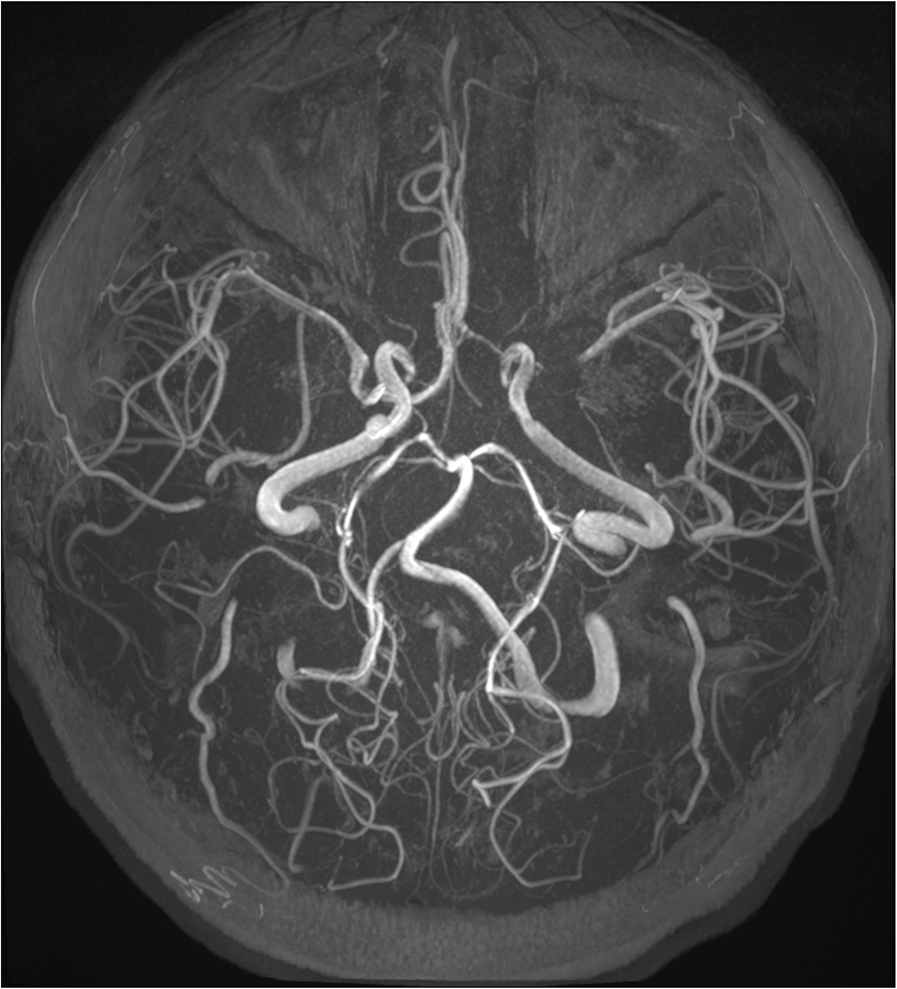
MRA axial view showing the presence of multiple stenoses of the arteries of the circle of Willis

**Fig. 4:**
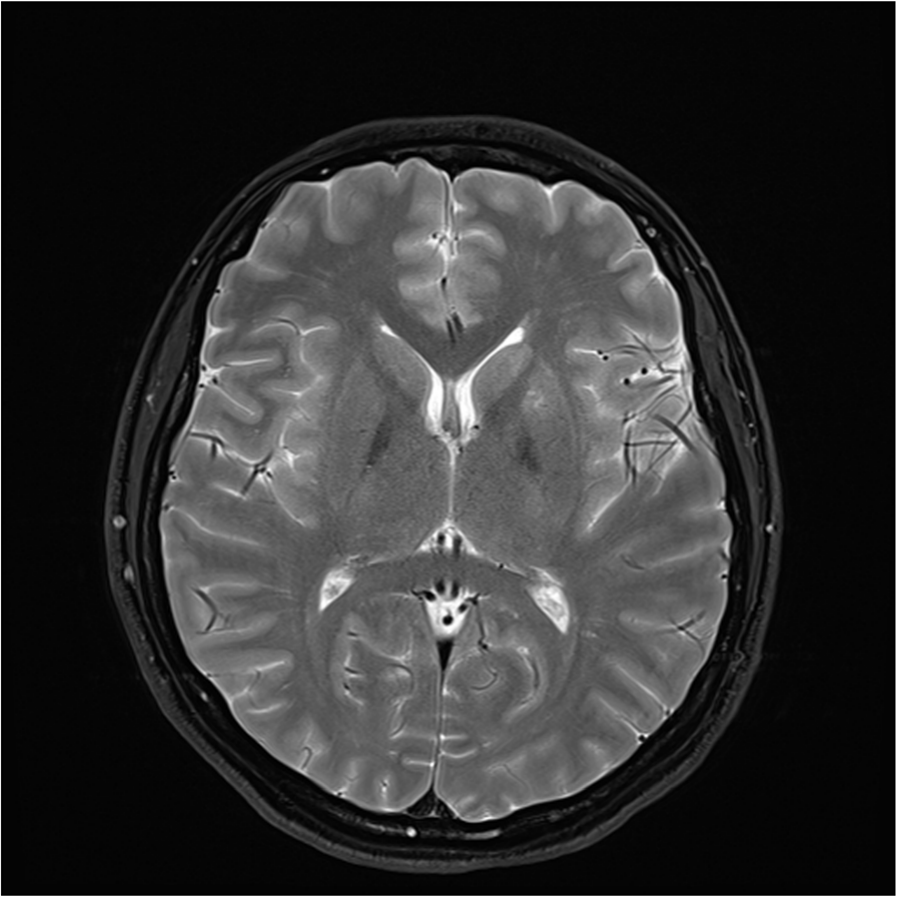
Cerebral MRI axial T2 sequence depicting bilateral engorged pial collateral vessels

Magnetic resonance angiography (MRA) (Figs. 2 and 3) and computed tomography angiography (CTA) of the supraaortic trunks showed subocclusive stenoses of both distal intracranial internal carotid arteries (ICA), M1 segment of the right middle cerebral artery (MCA), origin and medium third of A1 segment of the right anterior cerebral artery (ACA); significant hemodynamic stenoses of A1 segment of the left ACA, origin of the M2 segment of the left MCA and stenoses without hemodynamic significance of the proximal origin of the M1 and M2 segments of the left MCA, right M2 segment and of the origin of the left posterior cerebral artery (PCA) associated with the presence of engorged pial collateral vessels suggestive for MMV (Fig. 4). Although digital substraction angiography of the cervical and cerebral arteries is the gold standard for the diagnosis of MMD it could not be performed as the platelet number was constantly low.

To rule out a cardioembolic source for the disseminated ischemic lesions, we performed an electrocardiogram and 24-h-Holter monitoring that revealed sinus rhythm without any abnormalities. Moreover, echocardiography was normal in our patient.

Transcranial Doppler detection of high-intensity transient signal was normal but the vasomotor reactivity using Breath-holding index was impaired. Cervical arteries ultrasound excluded atherosclerosis.

Long-term electroencephalogram monitoring did not revealed abnormalities compatible with seizures.

Screening for nephritis and cataract, known to be associated with MYH9-RPD, by using abdominal ultrasonography, urine analysis, and ophthalmologic exam was negative.

Based on the characteristics of the episodes, the neurological exam, and the imaging findings that revealed the presence of multiple ischemic lesions and multiple stenoses of both distal ICA and of the arteries of the circle of Willis, the diagnosis of MMV associated with MYH9-related hereditary macrothrombocytopenia was established.

The patient received treatment with 80 mg daily of Verapamil with the disappearance of symptoms, with intensive monitoring of the blood pressure even by ambulatory blood pressure monitoring after release.

Two months later the patient presented one episode of dysarthria after a hot bath and few days later short episodes (2–3 min) of involuntary movements of the right upper limb.

Repeated cerebral MRI and CTA were similar to the previous ones, but long-term electroencephalogram monitoring depicted left frontal-temporal liminal epileptiform sharp waves.

The doses of Verapamil were increased and Levetiracetam was started, with resolution of symptoms.

The patient is scheduled to a 6-month follow-up in order to evaluate if other treatment interventions are needed.

An informed consent was obtained from the patient concerning the publication of this case report.

## Discussion and conclusions

In our patient the imaging findings established the diagnosis of MMV, which represents a different entity from MMD, and is characterized by an association with other diseases like atherosclerosis, irradiation, autoimmune, genetic, metabolic, hematologic or infectious diseases [4]. Thyroid disease is also associated with MMV with a higher prevalence in female patients. It is considered that high levels of thyroid hormones may lead to vascular abnormalities secondary to an impaired sensitivity to sympathetic stimuli but autoimmunity may also be involved in the pathogenesis of MMV [2].

Although a lot of conditions are associated with MMV, it is difficult to establish if the association is causal or incidental, due to the limited number of patients [4]. The association with hematologic conditions involving the platelets is very rare. There are a couple of cases described in the literature of patients with essential thrombocytemia or immune thrombocytopenia and MMV [5, 6]. The association of MMV with MYH9-RPD is uncommon. Only a few cases described in the literature presented with intracranial haemorrhage [7]. Best to our knowledge, there is no reported case with the MMV, MYH9-RPD and ischemic stroke.

The pathogenic link between MMS and MYH9-RPD is not very clear. Thrombotic events in these patients are probably related to arterial wall changes associated to the abnormal volume and function of the platelets [8]. Even the pathophysiological mechanisms of MMD are unknown, but a genetic contribution is incriminated and supported by recent molecular data. In some instances, identification of new genes led to the recognition of new pathways involved in the development of MMS. Recent researches found an overexpression of proangiogenic factors in different samples (cerebrospinal fluid, blood, tissues) collected from the patients, which supports the hypothesis that abnormalities in the angiogenesis are somehow involved in MMS pathogenesis. However, there is still a matter of debate whether this is primary process or secondary to cerebral ischemia [4, 9].

Beside typical imaging findings of MMD our patient presented stenoses of the second segment of ACA, MCA and PCA. Although MMD usually involves the proximal segments of MCA and ACA, additional stenoses of M2 segment of MCA were described in the literature in patients with MMV [10–12]. MMD was initially described involving the anterior circulation, but posterior circulation may also be affected [13].

MYH9-RPD is a group of autosomal dominant, X linked or recessive syndromes consisting in May-Hegglin anomaly, Sebastian syndrome, Fechtner syndrome, and Epstein syndrome. Thrombocytopenia is linked to a mutation in the MYH9 gene that encodes for nonmuscle myosin heavy chain IIA that is expressed in platelets and several other tissues. To date, 31 mutations of this gene associated with macrothrombocytopenia have been identified. Although the patients have a low platelet count, their function is normal or slightly impaired [3, 8].

Bleeding diatheses are known to be present in patients with MYH9-RPD but thrombotic events are rare, and there are only few case reports of patients with ischemic strokes but without MMV [14]. Some researchers considered that increased platelet volume correlates with a reduced risk of bleeding and a higher risk of thromboembolic events, but there are insufficient data to support this hypothesis [15].

Each disorder of this group is characterized, beside thrombocytopenia, by a combination of clinical and laboratory findings like granulocyte inclusion bodies, hypoacusia, glomerular nephropathy, and cataract [16]. In our patient, nephritis and cataract were excluded by clinical and ancillary laboratory tests, and leukocyte inclusion bodies were absent. She presented only hearing loss, as some genetic variants are associated mainly with sensorineural hypoacusia secondary to cochlear-saccular degeneration [16].

There are a lot of controversies concerning the treatment of MMD. Nowadays, cerebral revascularization through different surgical techniques is the treatment of choice [17], but this was not an option for our patient due to the low platelet count, which would increase the risks of the surgery.

Secondary prevention in patients with MMV is centered on treating the underlying pathology but in the case of our patient the haematologist did not recommend any treatment.

The use of antiplatelet drugs in MMD is still subjected to debate, as these drugs did not prove the expected efficacy. In a study published in 2018, Oki et al. observed that Aspirin was the most frequently used antiplatelet drug, followed by Cilostazol and Clopidogrel [18]. Due to an increased risk of bleeding, antiplatelet therapy was not initiated in our patient. We started the treatment with calcium channel blocker, Verapamil, which proved efficient so far. This class of drugs may reduce the frequency and severity of refractory transient ischemic attacks but there is no evidence that it could reverse or stabilize the progression of MMV [19]. Moreover, it is to be mentioned that its use is controversial due to its hypotensive effect which could precipitate cerebral ischemia, especially in a patient with impaired vasomotor reactivity [20]. In our patient, the chosen dosage of verapamil did not determined a decrease in blood pressure.

This is an interesting case of a patient with two rare pathologies, who presented with cerebral ischemic strokes. To our knowledge there are few cases described in the literature presenting with cerebral hemorrhagic events but none of them with multiple cerebral ischemic lesions. As these cases are very rare, it is important to gather evidence regarding the best approach and treatment strategy.

## Data Availability

All data generated or analysed during this study are included in this published article.

## Abbreviations

ACA: Anterior cerebral artery
CTA: Computed tomography angiography
ICA: Internal carotid artery
MCA: Middle cerebral artery
MMD: Moyamoya disease
MMS: Moyamoya syndrome
MMV: Moyamoya-like vasculopathy
MRA: Magnetic resonance angiography
MRI: Magnetic resonance imaging
MYH: Myosin heavy chain
MYH9-RPDs: Myosin heavy chain 9-related platelet disorders
PCA: Posterior cerebral artery
